# Interprofessional geriatric assessment in nursing home (IgAP): a curricular development in geriatrics

**DOI:** 10.3205/zma001528

**Published:** 2022-02-15

**Authors:** Navina Röcker, Birgit Wershofen, Yvonne Pudritz, Martin R. Fischer, Marc Auerbacher, Monika Fintz, Michael Drey, Ralf Schmidmaier

**Affiliations:** 1 LMU Klinikum, Ludwig-Maximilians-Universität (LMU) München, Medizinische Klinik und Poliklinik IV, Schwerpunkt Geriatrie, Munich, Germany; 2 LMU Klinikum, Ludwig-Maximilians-Universität (LMU) München, Institut für Didaktik und Ausbildungsforschung in der Medizin, Munich, Germany; 3 LMU Klinikum, Ludwig-Maximilians-Universität (LMU) München, Apotheke des LMU Klinikums & Department Pharmazie-Zentrum für Pharmaforschung an der LMU, Munich, Germany; 4 LMU Klinikum, Ludwig-Maximilians-Universität (LMU) München, Poliklinik für Zahnerhaltung und Parodontologie, Munich, Germany; 5 LMU Klinikum, Ludwig-Maximilians-Universität (LMU) München, Staatliche Berufsfachschule für Pflege am LMU Klinikum, Munich, Germany

**Keywords:** medical education, geriatrics, curriculum, learning objectives, interprofessionalism

## Abstract

The hallmark of medical action in geriatrics is the interprofessional treatment of the patient by a multi-professional team consisting of doctors, nurses and therapists with the aim of treating the patients primarily in a way that preserves their function and thereby enabling them to live as independently as possible. Therefore, at the beginning of every geriatric treatment, there is a multiprofessional geriatric assessment of functional abilities. With regard to successful medical action, this necessarily requires all health professions involved to understand geriatric patients and their limitations. Under ideal circumstances, their competencies overlap. From the point of view of the related disciplines, this means to teach working together with the other professions – interprofessionally – and learning from one another in order to effectively collaborate. After comparing the existing education in geriatrics within the Medical Curriculum Munich (MeCuM) with the European catalog of learning objectives for geriatricians (UEMS-GMS), a deficit with regard to geriatric assessment was recognized in the field of multi-professional training. Therefore, the existing geriatric curriculum of the Ludwig Maximilians University (LMU) in Munich should be expanded to include an interprofessional course on geriatric assessment. This project report aims to show the development and implementation of this course. For this purpose, the model for curriculum development according to Kern was used by the planners to establish an interprofessional briefing. Due to its innovative character, the course received public recognition and is the basis for the expansion of interprofessionalism in the sense of professional cooperation in geriatrics. Establishing interprofessionalism in other disciplines and locations is welcome.

## Zusammenfassung

Kennzeichen medizinischen Handelns in der Geriatrie ist die interprofessionelle Behandlung des/der Patient*in durch ein multiprofessionelles Team bestehend aus Ärzt*innen, Pflegenden und Therapeut*innen mit dem Ziel, die Patient*innen vorrangig funktionserhaltend zu behandeln und dadurch ihre möglichst selbständige Lebensweise zu fördern. Deshalb steht am Anfang jeder geriatrischen Behandlung das multiprofessionelle geriatrische Assessment der Funktionsfähigkeiten. Das fordert im Hinblick auf erfolgreiches medizinisches Handeln notwendigerweise von allen beteiligten Gesundheitsberufen ein Verständnis für den älteren Menschen mit seinen Einschränkungen. Unter idealen Umständen überschneiden sich ihre Kompetenzen. Aus Sicht der zugehörigen Disziplinen bedeutet dies zu lehren, gemeinsam mit den anderen Professionen – interprofessionell – zu arbeiten und voneinander zu lernen im Sinne einer effektiven Kollaboration. Nach Abgleich des bestehenden Medizinischen Curriculums München (MeCuM) für das Fach Geriatrie mit dem europäischen Lernzielkatalog Geriatrie (UEMS-GMS) wurde im Bereich der multiprofessionellen Ausbildung ein Defizit im Hinblick auf das geriatrische Assessment erkannt. Daher sollte das bestehende Geriatriecurriculum der Ludwig-Maximilians-Universität (LMU) München um einen interprofessionellen Kurs zum geriatrischen Assessment erweitert werden. In diesem Projektbericht soll die Entwicklung und Implementierung dieses Kurses dargestellt werden. Dazu wurde das Modell zur Curriculumsentwicklung nach Kern zur Etablierung eines interprofessionellen Briefings von den Planer*innen angewandt. Aufgrund seines innovativen Charakters erfuhr der Kurs öffentliche Anerkennung und ist Basis für den Ausbau von Interprofessionalität im Sinne von beruflicher Zusammenarbeit in der Geriatrie. Interprofessionalität in weiteren Disziplinen und Standorten zu etablieren, ist begrüßenswert.

## Introduction

In times of demographic change, it is a societal and medical challenge to create a structure that allows old, multimorbid people to live as autonomously as possible despite disabilities and functional restrictions. This is what modern geriatrics stands for [1]. It is the medical special discipline that deals with physical, psychological, functional and social aspects in the medical care of older adults (EUGMS definition) [2]. The main goal is to optimize the functional status with an improvement in the quality of life and independence from external help. In order to be able to define achievable, individual goals of treatment, each geriatric care begins with a multi-professional geriatric assessment, an examination using standardized procedures by representatives of the respective health professions that are formally independent from eachother [3], [4]. In the interprofessional team, the multi-professional therapy concepts are determined on the basis of this, for the successful implementation of which cooperative, respectful and effective action in the team is essential [5]. In terms of sustainable training in geriatrics, it is essential to develop a multi-professional understanding of the geriatric patient and his/her possible limitations at an early stage. Geriatrics has been anchored in medical studies as a cross-sectional subject “medicine of aging” since the changes to the Medical Licensing Regulations (ÄAppO) in 2002. For student teaching in the geriatrics department, a European catalog of learning objectives has been available since 2014, based on a first concept paper from 2003 by the European Union of Medical Specialists – Geriatric Medicine Section (UEMS-GMS) [6]. The existing geriatric teaching in the clinical curriculum of LMU Munich was compared in December 2014 with the EU catalog of learning objectives with regard to the “minimum requirements” formulated therein. Recognizing deficits in relation to the aspect of multi-professional geriatric assessment, the *Interprofessional Geriatric Assessment in nursing home (“Pflegeheim”)* (IgAP) course was developed as an elective curriculum in human medicine. This should be a joint teaching unit of different health professions, which in the interprofessional team provide complementary and interlocking services to geriatric patients on an equal footing.

The planning of the IgAP concept was based on the curriculum deveoplent steps proposed by Kern et al. and integrated into the interdisciplinary basic year for clinical medicine (6^th^/7^th^ semester) [7]. This project report demonstrates the structured development process of the IgAP course, following the steps of problem identification, needs analysis, learning goal definition, selection of appropriate teaching methods including implementation and evaluation. The transparent presentation is intended to motivate other locations to offer similar courses.

## Description of the project

### Step 1: Problem identification and general needs assessment

The changes in age and social structure are leading to a marked increase in medical care needs and problems among geriatric patients. Characteristic for this change of life reality is a vulnerable patient group, in the sense of multimorbid patients with multiple medication [8]. Whileit is possible in the clinical context, be it inpatient or outpatient, to respond to the growing number of geriatric patients with needs-adapted care structures [1], it is far less established to examine geriatric patients where they live – primary care in the literal sense! According to the latest report from the German Center on Aging, 4.3% of people aged 65 and older received care in residential care facilities in 2017 [9]. With age, the proportion of people in need of inpatient care facilities rises sharply. This demographic trend necessitates complex health care services, for which professional qualifications, effective communication, and smooth interprofessional collaboration are essential [10]. The need for geriatric competence in all health professions requires innovative teaching approaches. It is recommended to promote interprofessional collaboration and to increasingly integrate practice-oriented learning settings and digital media into training programs [11], [12], [13]. 

The subject of geriatrics is part of the current ÄAppO. With regard to medical training, §1 of the current ÄAppO states that it should be “conducted in a practice- and patient-oriented manner” and that collaboration with members of other health care professions should be promoted [https://www.gesetze-im-internet.de/_appro_2002/BJNR240500002.html]. Likewise, interprofessional collaboration is required as a training objective in the Act on the Reform of the Nursing Professions [14]. On an interdisciplinary level, the core curriculum of medical education, the National Competency-based Learning objectives catalog for Medicine (NKLM) (2015) [15] also takes a stand on geriatrics and addresses it as a population medicine concern that requires distinguishing geriatric patients from other groups. 

At LMU’s Medical Faculty, interprofessional teaching has been systematically developed and tested step by step since 2014 as a series of elective courses. So far, interprofessional case discussions between nursing students and medical students including interprofessional ward round simulation [16], a summer school between master students of public health and epidemiology together with medical students [17], a clinical pharmacy course for pre-registration students of pharmacy and medicine [18], and an interprofessional escape room [19] have been offered. Interprofessional training wards in neurology and geriatrics are in the planning stage. These interprofessional curricular mosaic stones address a number of partly overlapping learning objectives from the NKLM and are to be further developed into a longitudinal interprofessional curriculum in order to make the specific competences of each individual profession useful for the patient [20]. To date, all courses have been offered in the Faculty's Centers for Teaching and Studies (ZeUS) and on selected wards. So far, there are no teaching offers in nursing homes or in rehabilitation facilities at the LMU. 

Due to the changing demands on health care, the differentiation and profiling between the training professions is also increasing [21]. Direct teaching in the context of geriatric and nursing home care offers the various professions an opportunity, which has not yet been systematically used, to become aware of their strengths and weaknesses in geriatric treatment in order to create synergies in the care team and to avoid medical treatment errors [22]. 

Ideally, geriatric assessment is a multidimensional, interprofessionally supervised process [23]. Lack of teaching of this primarily affects patients. Medical errors, such as incorrect drug administration, jeopardize patient safety [24]. In addition, geriatric assessment creates a certain expectation on the part of the patient regarding the precision of the examination, which in turn affects patient satisfaction [25]. The students and young physicians themselves are also affected, as they know the concept of interprofessionality but do not know how to implement it [26]. Problems caused by misunderstandings in communication and cooperation then lead to the feeling of “failure in the team” [27]. If early teaching of interprofessional collaboration does not take place, a sense of rivalry between the different disciplines and health care professions may manifest itself [28]. The latter entails that examinations or services on the patient are potentially performed twice without consultation, which is uneconomical for payers and ties up resources inefficiently [29]. 

As the current interprofessional education at LMU is being developed and expanded in many ways, the curriculum planners at LMU created the course Interprofessional Geriatric Assessment in the Nursing Home (IgAP) to address the need of development for teaching in the care context of the nursing home. IgAP is a course offering from the geriatric care field that incorporates the practice of interprofessional collaboration, on-site in a nursing home. 

#### Step 2: Targeted needs assessment

Teaching in geriatrics takes place at the Medical Faculty in the form of lectures, seminars, exercises and bedside lessons. As part of bedside lessons, medical students take a geriatric assessment of day clinic patients without an interprofessional reference. Those responsible for curriculum planning noticed this deficit in comparison with the EU catalog of learning objectives according to Singler et al., which depicts a total of ten geriatric learning objectives of the German-speaking area [6]. In particular, they identified the potential for improvement in offering an interprofessionally oriented event outside the university, for example in a nursing home.

The target group of the project could be doctors of all specialties and care levels. However, since the recommendation applies in medical training to encourage interprofessional collaboration as early as possible, the common characteristic “before entering professional life” defines the target group of IgAP. In detail, this includes students of medicine (6^th^-8^th^ semester, prior knowledge of geriatrics from theoretical lecture), dentistry and pharmacy (both 8^th^ semesters, without previous knowledge) as well as trainees in nursing (2^nd^ third of training, prior knowledge according to basic training). Since there are no common teaching units across these four professional groups yet, they cannot draw on previous experiences as a team in the interprofessional implementation of a geriatric assessment course.

The specific needs of the medical students were derived with the help of the NKLM, the catalog of learning objectives in geriatrics according to Singler et al. and detailed analysis of the geriatric topics taught so far in lectures and seminars at the MeCuM (Medical Curriculum Munich). While the topic “geriatric assessment” is taught at the level of competence level A (“subject-specific knowledge”) described in the NKLM in MeCuM, competence level B (“disease-related action competence”) cannot be achieved through the teaching format. Above all, this applies to the two according to Singler et al. defined learning objectives “ethics” and “interprofessional work” (competence fields see table 1 (Tab. 1)), for which there is no explicit teaching unit in the MeCuM. Under these boundary conditions, the following specific needs for medical students could be determined:

- The acquisition of professional knowledge [30] for geriatric assessment,
- acquiring the necessary communication skills to be able to work in a team (cf. NKLM, Chapter 8: “member of a team”),
- structured practical training in geriatric assessment in a team with representatives from other health professions with a focus on joint decision-making.

We decided on a blended learning approach [31] with a combination of online and face-to-face teaching in order to reach the different target groups with flexible learning opportunities and to prepare them for the joint practical phase. The inverted classroom method [32] provided a common basis through an initial web-based self-learning phase prior to the application of prior knowledge in the context of processing an interprofessional case. Group work on residents appears to be particularly suitable as a promising teaching format for developing interprofessional competence [33]. The depth of the knowledge transfer is limited by the time available (one afternoon). However, this timeframe best takes into account the needs of other interest groups, such as the nursing home residents themselves, and thus complies with the legal requirements (according to § 2 Para. 3 ÄAppO: “avoid unreasonable stress on patients from practical lessons [...]”).

#### Step 3: Goals and objectives

In order to improve the inadequate teaching of interprofessional care reality in geriatrics, a range of courses should be implemented in the curriculum with the overarching learning objective:

*The graduates of the MeCuM can perform a geriatric assessment in interprofessional teamwork and derive recommendations for action as a team.*

Subject-specific knowledge and skills already acquired for the geriatric stage of life may be advantageous, but is not a must, which is why the possibility of theoretical self-study should be given (factual knowledge). The course should show interprofessional cognition, skills and attitudes in geriatrics (action and justification knowledge) and enable communication and decision-making in a team (action competence). Attachment 1 lists the contents of the Interprofessional learning objectives (LZ 1.1 handling/LZ 1.2. dignity/LZ 2 health changes/LZ 3 brief anamnesis/LZ 4 ethical concepts/LZ 5 interprofessional work/LZ 6 care options/LZ 7 dangers) in detail and – if available – stating the reference to the UEMS-GMS. The attached table 2 (Tab. 2) follows the formulation of the subject-specific learning objectives for medicine, pharmacy, nursing and dentistry.

#### Step 4: Educational strategies

As already mentioned in step 2, the blended learning approach succeeds in raising all relevant target groups to the same level of knowledge in advance, so that this is the most suitable method for teaching in interprofessional contexts [https://lmu.casus.net]. Embedding an online unit in the course should also enable self-directed learning to prepare for the face-to-face phase [31]. For this purpose, the representatives of the professions involved in the geriatric assessment have developed a cross-professional case that is compulsorily processed in the CASUS online learning environment as an integral part of the course offer [34], [35]. In this online unit with a processing time of approx. 30 minutes, various teaching methods (exercise, self-test, self-study material, visualization, interactive video sequences) are used in various flash cards in order to do justice to the learning type of all participating students and at the same time structure the self-directed learning process [36]. The self-assessment instrument (exam format) for the tasks on the flash cards was designed in such a way that it specifically records the link between knowledge, practical skills and professional attitudes. As soon as the learners have worked on a task, they can compare their solution with the detailed expert answer online. Each exam format, including the MC questions, provides further information on expanding competencies.

The implementation of the interprofessional learning objectives follows a uniform scheme:

1. **Raising** awareness of the content of the learning objective
2. **activation** through individual learning success control
3. debriefing using a detailed **online expert response**.

Awareness was raised by using are photos, scientific articles, guidelines or models. Table 3 (Tab. 3) (see appendix) specifies the holistic structure of the online unit.

The classroom course completes the implementation of all learning objectives by:

1. The **practical implementation** of the assessment on nursing home residents in a nursing home
2. **feedback** on the recommendations for action developed (peer assessment)
3. **debriefing **by the lecturers on site

**Figure 1:**
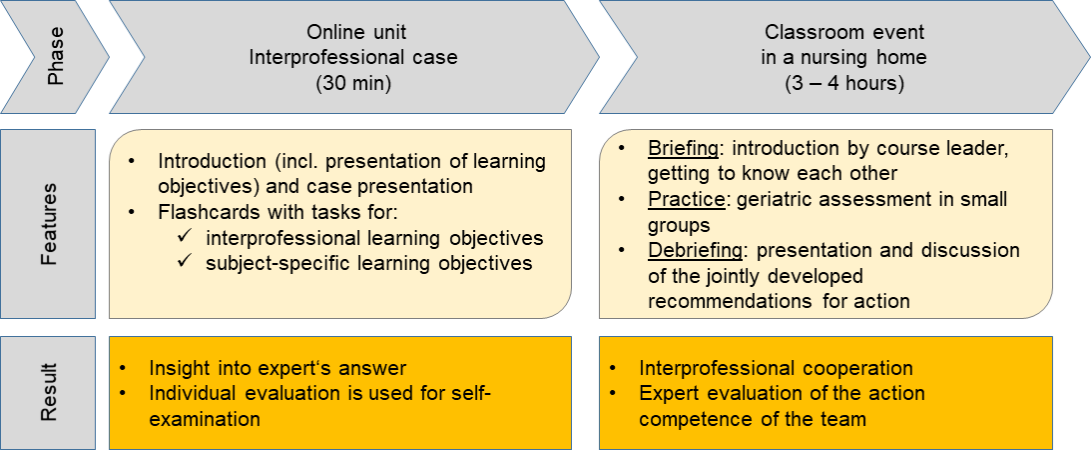
Schematic flow of the IgAP course

In summary, figure 1 (Fig. 1) gives anoverview of the course of IgAP.

An expert in interprofessional teaching is responsible for coordinating the face-to-face course, which takes place in the retirement and old people's home. In preparation for the attendance phase, the nursing staff select residents of the facility who would like to be available for an assessment. The residents resp. their supervisors confirm their written consent to participation. The seminar will be led on site by a team of four experts, including one representative from each of the health professions involved. The focus is on practically oriented learning objectives. After the briefing on the course of the seminar, the interprofessional groups of four students are created. Before the learners conduct their targeted surveys from the geriatric care sector, they have the opportunity to get to know each other. In order to achieve the team goal together, they are encouraged to contribute equally and to present a positive working atmosphere (mutual support, appreciation, result-oriented work, etc.). Following the assessment, the individual interprofessional small groups develop recommendations for action to improve the medical, nursing and social life situation on the basis of the data they have collected. This clarifies the importance of collaboration to the learners. Different interests of the health professions with regard to residents’ further treatment must be clearly communicated in the team and brought to a consensus. If necessary, the support of the subject matter experts can be obtained. The results are presented to the plenum using a flipchart. The examination format of the face-to-face event follows the classic formative assessment. From the feedback and additions from the other learners, such as the best possible care for the resident, it is possible to draw conclusions about the classification of the individual performance. Finally, the feasibility of the proposed recommendations is discussed and reflected upon with peers, experts and the nursing staff (debriefing).

#### Step 5: Implementation

The teaching project planned as an innovation should be part of the interdisciplinary basic year (modules 2 & 3) and be carried out once per semester. Modules 2 & 3 are the core element in clinical MeCuM for training in important medical key competencies, for example the ability to work in a team or to deal with colleagues in a professional manner. Locating the IgAP course in modules 2 & 3 followed the basic idea of breaking down a subject as complex as geriatrics into so-called islands of work. In the sense of a continuous learning spiral, the basic skills imparted in module 2 & 3 will later be taken up and deepened in the special geriatric teaching in module 5.

The new course should also be tailored to the needs of medical students for special deepening of interests and should therefore be established as a so-called compulsory elective event (VObI: event for cross-organ block deepening of interests). The costs for the development work of the IgAP teaching concept could be off-set by successfully acquiring third-party funding from the Teaching @LMU funding program. The latter is the central concept for the further development of the quality of teaching and studies at the LMU, supported by the “teaching quality pact” from the federal and state governments.

Central to geriatrics is the question of how teachers can get sufficiently suitable patients for the teaching purposes of the assessment. Those from the inpatient area are unsuitable due to the acute illness. Furthermore, the clinical process of this group of patients interferes with the longer-lasting multiprofessional assessment. In contrast, the patients in the university outpatient departments are usually too healthy and any uncertainties as to whether there are enough people in need of geriatric treatment are not acceptable. Overall, the existing geriatric day clinic is too small for the teaching assignment. Anticipating these hurdles, the curriculum planners found the solution to the problem outside of the university, in the nursing home. Embedded in their familiar environment, the activities of daily life can be recorded there realistically and without time pressure.

In the concrete implementation of IgAP, it was important for the curriculum planners to create a safe learning environment for the learners. The interprofessional exchange of views on problems in basic geriatric care has the potential for conflict [23], [26]. This can be driven by hierarchical levels, general conflict management in the team or working conditions on the ward. The online case, which has to be processed in advance, gives the learners insights into the tasks and responsibilities of the other professions, making it easier for them to start working together. After each completed learning card, there is immediate and value-neutral feedback that is uniform across all professional groups. At the same time, there is a transfer of knowledge via the other professions.

The possibility to work on the learning case independently of time and place makes it easier to bring the different curricula together. The coupling of the different professions turned out to be a major implementation hurdle. The student semester times are not congruent with the theoretical and practical phases of the health and nursing staff, which makes it difficult to find an appointment for the practical unit.

The curriculum planners personally implement the requirement for high-quality teaching in the online and face-to-face phase of the course. The specialists from medicine (geriatrics), dentistry, pharmacy and nursing who were present at the nursing home also designed the relevant, subject-specific flash cards in CASUS individually as well as the content of the interprofessinal flash cards as a team.

#### Step 6: Results and evaluation 

The IgAP course has been offered at the LMU Medical Faculty since the winter semester of 2016 and has so far been completed by 105 students. From the beginning, the course was offered as a compulsory elective subject once per semester, so that it has taken place seven times to date. Initially, the course had a capacity of 16 learners, i.e. four learners for each discipline. While the target group definition also included physiotherapy trainees in the planning of the IgAP project and the participation of dentistry students was not guaranteed for the time being, its implementation phase showed that physiotherapy students could not participate for organizational reasons. Instead, the curriculum planners decided to consolidate the important topic of dental and oral hygiene in geriatrics in the course, so that the IgAP concept in its implemented form was further developed and supplemented to include dentistry students. In this way, the care facility also benefits from IgAP: a nurse who is present documents the results and passes them on to the responsible (dental) doctors on site or, if necessary, arranges an examination appointment for the residents [37].

In the 2019 summer semester, the course was expanded to include six interprofessional groups. On the basis of a well-founded proposal, including a letter of recommendation submitted by students, the IgAP course was awarded the teaching innovation prize in 2019. In particular, the high didactic quality was responsible for the award of the teaching award and the fact that the project thus picks up on a highly relevant socio-political discussion on more interprofessional cooperation [38].

In order to counter the high logistical and personnel effort, the existing case was modified as an e-learning case (online IgAP) for asynchronous learning with the help of the financial support received. A change in the overarching learning objectives only related to the suspension of direct interprofessional collaboration in the nursing home. Due to more detailed information and in-depth questions, the average processing time increases to approx. 60 minutes. This offer for self-study of an interprofessional geriatric assessment has been used by 397 learners so far. In the meantime, this case has become mandatory for medical students in modules 2 & 3 of geriatric teaching. Participation in the online IgAP is voluntary for the other professions. The prospect of including further health professions is planned, including the renewed involvement of physiotherapy students.

## Discussion and critical reflection

The IgAP course conveys important technical content and focuses on interprofessional work in the care context of an old people's and nursing home. The project development extended from the first idea at the end of 2015 to coordinating the teachers of all professions involved and the selection of appropriate teaching/learning methods up to the start of the course in the 2016/17 winter semester. The IgAP concept has already been presented at two educational congresses in the health sector and has also gained national recognition by winning the LMU 2019 Teaching Innovation Award. Interprofessional education is highly relevant for patient safety, understanding of roles, job satisfaction and cost reduction in the health system, but its implementation is also subject to a situational assessment.

Due to the trend to relocate care services from the inpatient to the outpatient area, the demands on the ability to cooperate and the communication skills of the individual health professions are increasing and a corresponding expansion of the skills of all those involved is necessary [39]. However, the strategy to achieve the goal – the provision of equally complementary and interlocking services – should be a project-related individual decision. It can be beneficial and even more sustainable to use the teach-the-trainer format in a curriculum project to implement interprofessional training at the same time as the bottom-up strategy (i.e. first addressing the lowest training level). In the first instance, the management levels would be qualified for interprofessional training so that the hierarchical levels below benefit from the transfer of knowledge.

In its current form, IgAP can be transferred to other practical learning settings, such as in outpatient care or rehabilitation and the inclusion of other health professions. Further follow-up projects could also focus on interprofessional collaboration in other disciplines, such as diabetology (collaboration between internist, neurologist, nutritionist and sports therapist).

## Conclusions

In summary, after a corresponding needs analysis, the course *Interprofessional Geriatric Assessment in Nursing Homes (IgAP)* was successfully implemented in the existing geriatric curriculum of Faculty of Medicine at LMU Munich using digital media. The course is characterized by its interprofessional character (human and dental medicine, pharmacy, and nursing) at a non-university learning location – for the first time a nursing home. The basis of the successful cooperation was the good preparatory work by the planners of the course, so that appropriate spatial, material, temporal and human resources could be provided for its implementation. The long-term goal of the IgAP team is to gradually expand the course. In response to the positive feedback from the initial concept, this has already been initiated by recording a second virtual learning case. With a view to the aging population, it is profitable to establish the multi-professional geriatric assessment as a standardized process with an interprofessional orientation at other locations as well. We hope that IgAP will also encourage other universities to take organizational and structural measures to improve interprofessional collaboration.

## Authors

The authors Michael Drey and Ralf Schmidmaier contributed equally in the creation of the manuscript. 

## Competing interests

The authors declare that they have no competing interests. 

## Supplementary Material

Structure and learning objectives of the online unit (= interprofessional case in CASUS) of the elective course IgAP

## Figures and Tables

**Table 1:**
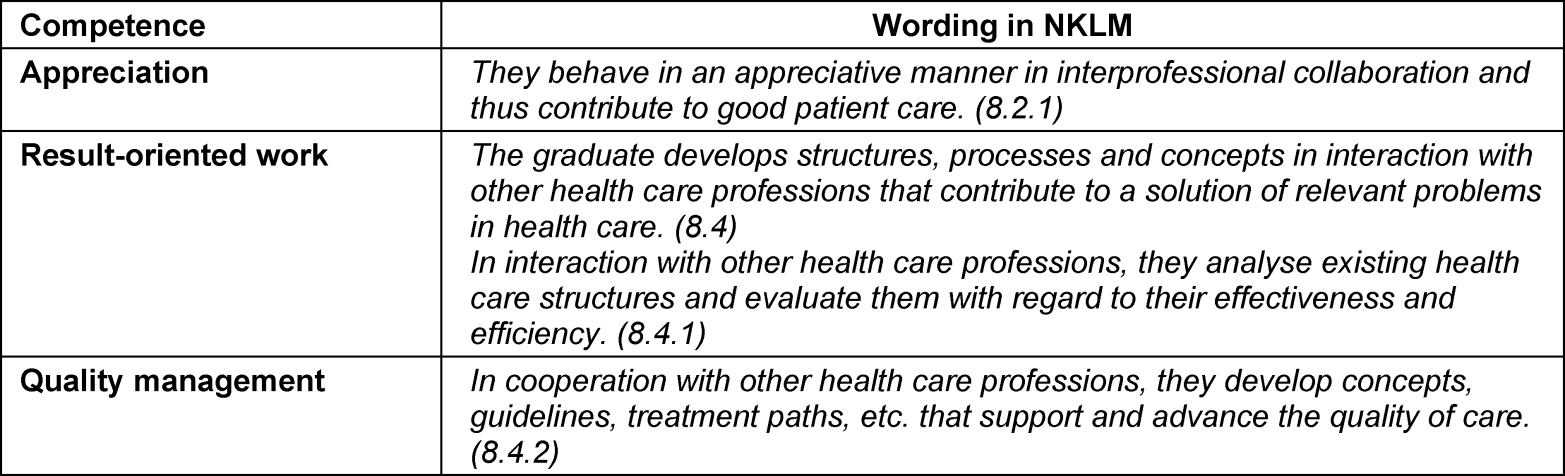
Central competence fields of interprofessional teamwork based on the NKLM

**Table 2:**
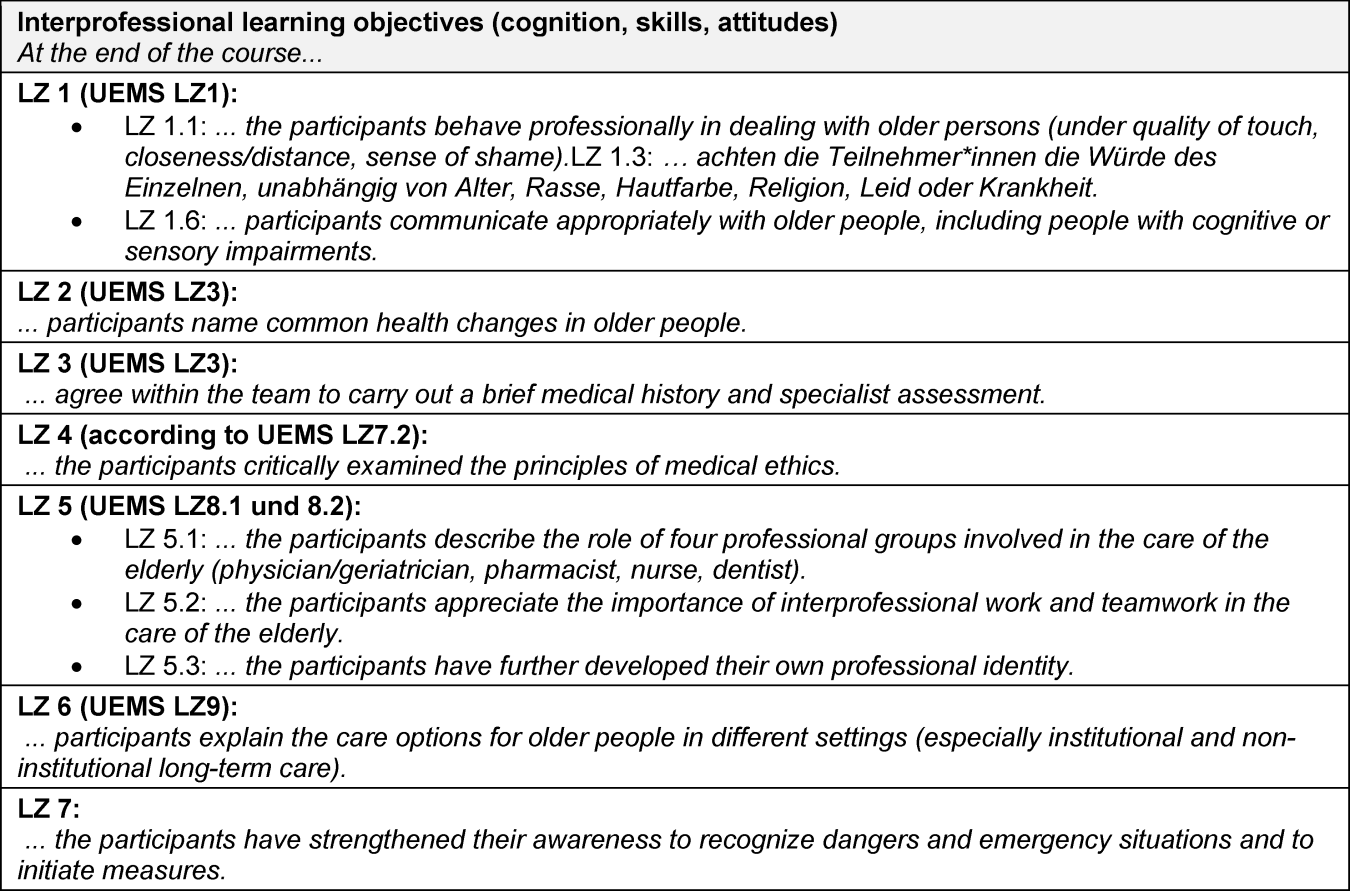
Interprofessional learning objectives of the IgAP course

**Table 3:**
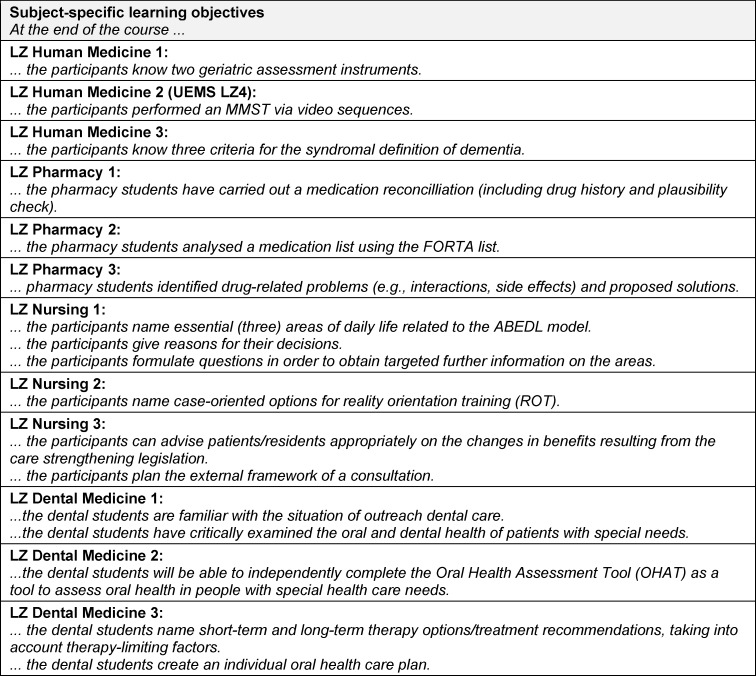
Subject-specific learning objectives of the IgAP course
