# Supplementary material for: Interprofessional geriatric assessment in nursing home (IgAP): a curricular development in geriatrics
Source: GMS J Med Educ. 2022 Feb 15;39(1):Doc7. doi: 10.3205/zma001528 (PMC8953190; doi:10.3205/zma001528)
Supplement: Structure and learning objectives of the online unit (= interprofessional case in CASUS) of the elective course IgAP [file JME-39-7-s-001.pdf]

Attachment 1: Structure and learning objectives of the online unit (= interprofessional case in CASUS) of the elective course IgAP.

	Flashcard	Learning Objectives	Content, method and self-assessment tool
Introduction	1: Welcome card		Welcome, agenda, handing out the learning objectives
	2: Experiences, Expectations		Questionnaire on cooperation between health care professions (10 items),<br>**Open ended question** about experiences and expectations and "how do you imagine the contact with the resident?"
	3: Storyboard		Case presentation: Mrs. Roth, 84 years old, has been living in the Münchenstift nursing home on Rümannstraße for three months ...
Interprofessionalism	4: LZ 1.1 Handling	Professional interaction with older persons	Sensitization on the subject of dealing with older persons, photo<br>**MC-Question:** "Professional interaction with the elderly and nursing home residents like Ms. Roth means you ..."
	5: LZ 1.2 Dignity	Respect for the dignity of the individual	Handout of the principle of work in the nursing home<br>**Cloze** on the dignity of the nursing home resident
	6: LZ2 Health Changes	Name common health changes that occur	Handout of a contribution on the subject of abilities and limitations of home residents<br>**MC-Question:** "What geriatric syndromes and problems may be causative for recurrent falls of unclear etiology?"
	7: LZ 3 Brief medical history	Carrying out a brief anamnesis in the team	Handout Guide Multidimensional Geriatric Assessment (Physical, Cognitive, and Mental).<br>**Assignment question:** text modules on brief anamnesis, geriatric assessment and summary of results.
	8: LZ 4 Ethical concepts	Critical examination of ethical concepts	Model of the "Four Principles of Medical Ethics" according to Beauchamps/Childress (Autonomy, Beneficence Principle, Nonmaleficence Principle, Justice).<br>**Open ended text field:** Imagine a medical challenge with corresponding ethics.
	9: LZ 5 Interprofessional work	Reflection on and implementation of interprofessional work (role, respect, own identity))	Raising awareness on the topic of interprofessional cooperation and visualization by means of a photo symbolically representing each professional group<br>**Open ended question:** consider the roles of the professions involved and "describe the roles in terms of tasks that these professions perform."
	10: LZ 6 Care options	Explain institutional and non-institutional care options.	The Münchenstift nursing home as an example of an environment of institutional long-term care<br>**Underlining task:** "What other ways of providing care might be relevant?"
	11: LZ 7 Dangers	Recognition of dangers and emergency situations	Listing meassures to prevent falls and fall-related injuries<br>**Assignment question:** keywords on intrinsic and extrinsic risk factors.
	12: Info about the subject specific learning objectives		Encouraging students to also work on the flashcards of the other professions

Attachment 1 to Röcker R, Wershofen B, Pudritz Y, Fischer MR, Auerbacher M, Fintz M, Drey M, Schmidmaier R. *Interprofessional geriatric assessment in nursing home (IgAP): a curricular development in geriatrics.* GMS J Med Educ. 2022;39(1):Doc7. DOI: 10.3205/zma001528

**Human Medicine**	13: Human Medicine 1	Know two geriatric assessment tools	Systematic recording of limitations of the patient's cognition and mood in the nursing home, output of the Mini-Mental Status Test (MMST) and the Geriatric Depression Scale (GDS).<br>**Open ended question:** "What is the name of the assessment for cognition or mood?"
	14: Human Medicine 2	Carrying out an MMST	Paraphrase of dementia as a clinical syndrome with disturbances of many higher cortical functions, repeated output of the MMST as well as eleven video sequences (each about 30 seconds long) demonstrating test performance on the patient.<br>**MC-Question:** Which prompts has the patient correctly complied with and what is the severity of dementia with them?
	15: Human Medicine 3	Know three criteria of syndromal definition of dementia.	Handout the S3 Guideline "Dementias" (Long version)<br>**Open ended question:** "Do you know the three criteria for the syndromal definition of dementia?"
**Pharmacy**	16: Pharmacy 1	Carrying out a medication reconcilliation	Raising awareness of the importance of pharmaceutical care in the home (holistic!), obligation to take a medication history, issuing the plausibility checklist: Medication Appropriateness Index (MAI).<br>**Prioritization question (sort answer):** "Rank the following plausibility check items in order of relevance."
	17: Pharmacy 2	Analysis of a medication list for potentially inappropriate medication (PIM)	Awareness of benefit assessment of geriatric medication, issuing of FORTA list as well as patient's medication plan.<br>**Assignment question:** "Classify the patient's medications according to the FORTA list."
	18: Pharmacy 3	Identification of drug-related problems and proposal for solutions	Sensitization to drug-related problems (DRP), handout of summary of product characteristic regarding citalopram.<br>**MC-question:** Which of the following statements are relevant proposed solutions to the patient's potential drug-related problems?"
**Nursing**	19: Nursing 1	Naming and understanding essential areas of daily living according to ABEDL model.	Structured elicitation of nursing care needs, output of the ABEDL structuring model (basic nursing principles) as well as sample basic nursing assessment<br>**MC-Question:** Name the areas of daily living in which the patient is most likely to need caregiver support based on the ABEDL model.<br>**Reason your answer.**
	20: Nursing 2	Name case-oriented options for reality orientation training (ROT)	Target definition of the reality orientation training (ROT)<br>**Open ended question:** "To what extent can ROT be applied to the patient's room design?"
	21 / 22 / 23 / 24 / 25: Nursing 3	Appropriate advice on benefit changes due to care strengthening legislation	Story about changes in the health care system, issue of the care strengthening laws (Pflegestärkungsgesetze) as well as information on how to prepare a consultation (checklist).<br>**Open ended question:** Contact to request an assessment, criteria for the assessment, legal basis for the renewals, information about the amount of the co-payment, self-reflection on one's own area of competence of the counseling interview, five central aspects about the framework of the counseling interview
**Dentistry**	26: dental medicine 1	Know geriatric assessment tools from dentistry.	Systematic assessment of dental and oral health, issuance of the Oral Health Assessment Tool (OHAT), and background information on oral assessment tools.
	27: dental medicine 2	Name causes of food refusal and halitosis	Food refusal and halitosis<br>**Open ended question:** "What are the possible causes of food refusal from a dental point of view? What can the halitosis be due to?"

	28: dental medicine 3	Create brief dental findings and name short-term and long-term treatment options/treatment recommendations.	Photo and description of the oral hygiene situation<br>**Single look diagnosis with open ended text response:** perform a dental examination, diagnostics, therapy (short- and long-term), medication risk management, individual oral health plan
**Closing**	29: Conclusion		Summary Interfaces of the professions involved, questionnaire for the evaluation of the online course IgAP.<br>**Open ended question:** "How do you help ensure that constructive interprofessional collaboration can occur? Why is this important?"
	30: end		Greeting and farewell of the IgAP team management

Attachment 1 to Röcker R, Wershofen B, Pudritz Y, Fischer MR, Auerbacher M, Fintz M, Drey M, Schmidmaier R. *Interprofessional geriatric assessment in nursing home (IgAP): a curricular development in geriatrics.* GMS J Med Educ. 2022;39(1):Doc7. DOI: 10.3205/zma001528
